# Ways of improving the precision of eye tracking data: Controlling the influence of dirt and dust on pupil detection

**DOI:** 10.16910/jemr.10.3.1

**Published:** 2017-05-25

**Authors:** Wolfgang Fuhl, Thomas C. Kübler, Dennis Hospach, Oliver Bringmann, Wolfgang Rosenstiel, Enkelejda Kasneci

**Affiliations:** University of Tübingen, Germany

**Keywords:** eye tracking, pupil detection, robustness, dirt simulation, data quality

## Abstract

Eye-tracking technology has to date been primarily employed in research. With recent advances in affordable video-based devices, the implementation of gaze-aware smartphones, and marketable driver monitoring systems, a considerable step towards pervasive eye-tracking has been made. However, several new challenges arise with the usage of eye-tracking in the wild and will need to be tackled to increase the acceptance of this technology. The main challenge is still related to the usage of eye-tracking together with eyeglasses, which in combination with reflections for changing illumination conditions will make a subject "untrackable". If we really want to bring the technology to the consumer, we cannot simply exclude 30% of the population as potential users only because they wear eyeglasses, nor can we make them clean their glasses and the device regularly. Instead, the pupil detection algorithms need to be made robust to potential sources of noise. We hypothesize that the amount of dust and dirt on the eyeglasses and the eye-tracker camera has a significant influence on the performance of currently available pupil detection algorithms. Therefore, in this work, we present a systematic study of the effect of dust and dirt on the pupil detection by simulating various quantities of dirt and dust on eyeglasses. Our results show 1) an overall high robustness to dust in an offfocus layer. 2) the vulnerability of edge-based methods to even small in-focus dust particles. 3) a trade-off between tolerated particle size and particle amount, where a small number of rather large particles showed only a minor performance impact.

## Introduction

With the advent of affordable eye-tracking technology to consumer products like controllers for video gaming, interaction with smartphones, or driver monitoring, new challenges arises. Outside of the controlled conditions of a laboratory, a reliable eye-tracking can hardly be achieved. The main source of error in such settings is a non-robust pupil signal which primarily arises from challenges in the image-based detection of the pupil due to changing illumination, especially for subjects wearing glasses. Excluding such subjects or declaring a customer as untrackable (commonly 5-10% in lab setups Schnipke and Todd ( 11 )) is not an option anymore. Instead, customers expect eye-tracking to just work. Hence, relia-bility of the eye-tracking signal is still an important issue.

One of the first data processing steps for video based eye-tracking is the localization of the pupil within the eyetracker image. Benchmark data for pupil detection are declared especially challenging (and in fact are) if people are simply walking around outdoors or driving a car Fuhl, Santini, Kübler, and Kasneci ( 4 ); Fuhl, Tonsen, Bulling, and Kasneci ( 5 ); Tonsen, Zhang, Sugano, and Bulling ( 14 ). However, data quality means much more than the mere tracking rate Holmqvist, Nyström, and Mulvey ( 6 ), yet it certainly is amongst the most fundamental factors. A tracking loss affects all subsequent processing steps, such as calibration and fixation identification. Therefore, it alters almost every key metric used in eye-tracking research Wass, Forssman, and Leppänen ( 15 ) and can be extremely frustrating during interaction with a device.

The development of robust algorithms has to keep pace with the availability of consumer devices. In order to improve the current generation of algorithms, we need to get a better understanding of the factors that cause a decrease of tracking quality in real-world applications.

In this work, we systematically study the impact of dust and dirt on the tracking rate. As most of today’s eyetrackers are video based, dirt and smudges, both on the device as well on the subject’s eyeglasses, are a potential source of error that may be less common in a well maintained laboratory, but become relevant in real-world applications. Just think of a remote tracking setup in an automotive driver monitoring system. Since it is hard to objectively quantify the amount and nature of dirt in a real experiment, we employ an image synthesis method on top of real eye-tracking videos recorded during a driving experiment. Tracking rate and performance of four state-of-the-art pupil detection algorithms, namely Świrski and Dodgson ( 13 ), ExCuSe Fuhl, Kübler, Sippel, Rosenstiel, and Kasneci ( 2 ), Set Javadi, Hakimi, Barati, Walsh, and Tcheang ( 7 ), and ElSe Fuhl, Santini, Kübler, and Kasneci ( 4 )are evaluated.

The remaining of this paper is organized as follows. The next Section gives an overview over the competing pupil detection algorithms and discusses related work in image synthesis for eye tracking. Details on the particle simulation are given in Section Methods. Section Results presents the performance of the state-of-the-art pupil detectors for various conditions. Finally, the obtained results are discussed and conclusions are drawn.

## Related work

### Pupil detection algorithms

Although many commercial eye-tracker manufacturers do not provide exact documentation of their pupil detection method, there are a number of published algorithms. In the following, we will provide a summary of the workflow for a selection of algorithms. For a more detailed overview and comparison of the state-of-the-art we refer the reader to a recent review by Fuhl, Tonsen, et al. ( 5 ). In the following we will briefly discuss details of some of these algorithms, namely Świrski, Bulling, and Dodgson ( 12 ), Else Fuhl, Santini, Kübler, and Kasneci ( 4 ), and ExCuSe Fuhl et al. ( 2 )due to their good performance in prior evaluations Fuhl, Tonsen, et al. ( 5 ) and their conceptual differences. ElSe Fuhl, Santini, Kübler, and Kasneci ( 4 )was chosen as the currently best performing state-of-the-art method Fuhl, Geisler, Santini, Rosenstiel, and Kasneci ( 1 ). We also include the Set algorithm Javadi et al. ( 7 ) as a representative of simple, threshold-based approach.

### Algorithm ExCuSe

The Exclusive Curve Selector (ExCuSe)Fuhl et al.( 2 )first analyzes the image based on the intensity histogram with regard to large reflections. For images with such reflections, the algorithm tries to find the pupils outer edge, otherwise this step is skipped. To localize the pupil boundary, a Canny edge filter is applied and all orthogonally connected edges are broken at their intersection. This is done by applying different morphologic operations. All non-curvy lines are then removed. For each curved line, the average intensity of its enclosed pixels is computed. The curved line with the darkest enclosed intensity value is selected as pupil boundary candidate and an ellipse fit is applied to it. If the previous step did not yield a clear result or was skipped, a binary threshold based on the standard deviation of the image is applied. For four orientations, the Angular Integral Projection Function Mohammed, Hong, and Jarjes ( 10 ) is calculated on the binary image and an intersection of the four maximal responses is determined. This intersection location is further refined within the surrounding image region by using similar or darker intensity values as attractive force. Based on the refined position, the surrounding image region is extracted and a Canny edge filter applied. These edges are refined using the binary image obtained by applying a calculated threshold. Beginning at the estimated center location, rays are send out to select the closest edge candidates. The last step is a least squares ellipse fit on the selected edges to correct the pupil center location.

### Algorithm ElSe

The Ellipse Selector (ElSe) Fuhl, Santini, Kübler, and Kasneci ( 4 )begins by applying a Canny edge filter to the eye image. Afterwards, all edges are filtered either morphologically or algorithmically. In this work, we used the morphological approach due to the lower computational demands. The filter removes orthogonal connections and applies a thinning and straightening with different morphologic operations than ExCuSe Fuhl et al.( 2 ). Afterwards, all straight lines are removed and each curved segment is evaluated based on the enclosed intensity value, size, ellipse parameters, and the ease of fitting an ellipse to it. The last evaluation metric is a pupil plausibility check. In case the previously described step fails to detect a pupil, a convolution based approach is applied. Therefore, a mean circle and a surface difference circle are convolved with the downscaled image. The magnitude result of both convolutions is then multiplied and the maximum is selected as pupil center estimation. This position is refined on the full sized image by calculating a intensity range from its neighborhood. All connected pixels in this range are grouped and the center of mass is calculated.

### Algorithm Set

Set Javadi et al. ( 7 )can be subdivided into pupil extraction and validation. An intensity threshold is provided as a parameter and used to convert the input image into a binary image. All connected pixels below (darker than) the threshold are considered as belonging to the pupil and grouped together. Pixel groups that exceed a certain size, provided to the algorithm as a second parameter, are selected as possible pupil candidates. For each such group the convex hull is computed and an ellipse is fit to it. This ellipse fit is based on comparing the sine and cosine part of each segment to possible ellipse axis parameters. The most circular segment is chosen as the final pupil.

### Algorithm by Świrski et al.

In a first step of the algorithm introduced by Świrski et al. ( 10 ), Haar-Cascade-like features of different sizes are used to find a coarse position for the pupil. To save computational costs this is done on the integral image. The range at which these features are searched is specified by a minimum and maximum pupil radius.

This results in a magnitude map where the strongest response is selected as coarse pupil center estimate. An intensity histogram is calculated on the surrounding region. This histogram is segmented using k-means clustering, resulting thus in an intensity threshold. This threshold converts the image into a binary pixel inside-pupil, outside-pupil image. The largest continuously connected patch is selected as pupil and its center of mass as the refined pupil center location. In the final step, an ellipse is fitted to the pupil boundary. A morphologic preprocessing by an opening operation is applied to the image to remove the eyelashes. Afterwards, the canny edge detector is used for edge extraction. Edges that surround the refined pupil location are selected and an ellipse is fitted using RANSAC and an image aware support function for edge pixel selection.

### Image synthesis in eye-tracking algorithm development

Each eye-tracking recording is associated with a quite unique mixture of noise components. Therefore, artificial eye models and image synthesis methods for eye-tracker images were created in order to produce mostly artifactfree recordings. Świrski and Dodgson ( 13 ) even model and render the complete head to generate data for remote as well as head mounted eye trackers. The model renders as physically correct as possible, including reflections, refraction, shadows and depth-of-field blur together with the facial marks like eyelashes and eyebrows.

Wood, Baltrušaitis, Morency, Robinson, and Bulling( 17 ) used rendered images for estimating the gaze of a person using a k nearest neighbor’s estimator. For fast rendering they employed the Unity game engine. Furthermore, the authors generated data for different skin colors, head poses, and pupil states. The accuracy of this concept was further improved by Zhang, Sugano, Fritz, and Bulling ( 18 ) using a convolution neuronal network trained on the complete face.

The work by Kübler, Rittig, Kasneci, Ungewiss, and Krauss ( 9 ) advances in a different direction. More specifically, the authors evaluate the effect of eyeglasses on traditional gaze estimation methods by including the optical medium into the simulation model. The authors showed that eyeglasses have a major impact on gaze direction predicted by a geometrical model, but not on that of a polynomial fit.

## Methods

### Observations on real recordings

**Figure 1:**
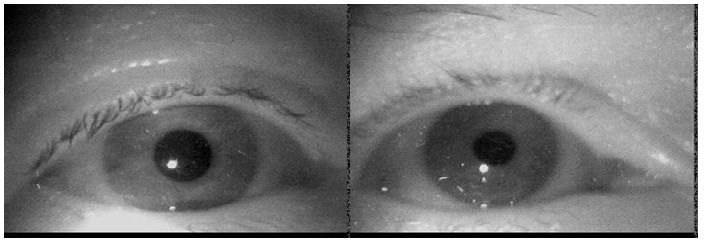
Example eye images of two subjects showing dust particles in the focus layer of the camera (because they are best visible in print). Most dust particles in our data were placed slightly outside of the focus layer and therefore blurred.

To get an impression of the impact of dust during realworld eye-tracking, we browsed about 30 datasets from a real-world driving experiment Kasneci et al. ( 8 ). Dust particles are almost invisible on still images, but become clearly visible in a video. This is due to the static behavior of dust while the eye is moving. Figure 1 shows some examples of dust we found in the dataset.

### Dirt particle image synthesis

In 2005, Willson et al. first described a method for the simulation of dust particles on optical elements in Willson, Maimone, Johnson, and Scherr ( 16 ). They formulated a camera model, derived the influence of dust particles on the final image, and specified formulae to calculate these effects. However, their particle model and the final image synthesis were still lacking some of the occurring effects: particles were modeled as circular achromatic shapes that were distributed on a plane perpendicular to the image sensor. We extended their work by modeling particles as triangulated objects with color and positions in 3D, which allows distribution in potentially arbitrary 3D-subspaces, e.g. curved and rotated planes. By modeling particles as a set of triangles and adding color information, the formulae for intersection calculation and color blending are significantly different and more expensive to compute. To calculate the final image in reasonable time, a rtree is used to speed the location of particles close to a specified point.

As presented by Willson et al. ( 16 ), the influence of dust particles on the final image depends on the camera model and the dust particle model. These models are presented in the following subsections.

### Camera model

**Figure 2:**
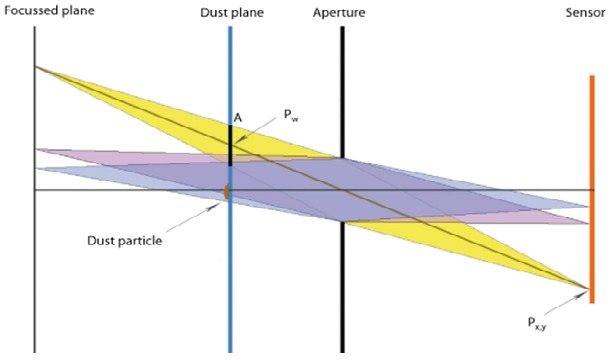
Camera model of the dust simulation. Notice the parameters A (area of the projected collection cone), *p_w_* (intersection point of the collection cone with the dust plane and *p_x,y_* (pixel position on the sensor)

In real cameras, an aperture controls the amount of light and the directions from which light is collected. This bundle of light rays is called the collection cone. Such a camera model that respects was employed in this work to vary the depth of field, to gain naturally blurred images of objects that are out of focus and control for the amount of light and blur, Figure 2.

### Dust particle model

We model dust particles based on position, color (including an alpha channel for transparency), shape variance, and size. They are randomly distributed on a userdefined plane, not necessarily perpendicular to the image plane. As shape we modify a basic circle by smooth deviations. The final shape is triangulated and the triangulation detail level controlled by specifying the number of edges for each particle. The maximum extent of the dust plane is calculated using the maximum angle of view of the camera. Finally, by setting the number of particles for the next simulation run, the desired particles are distributed over the given plane subset.Using the center of location *d_i_* of the dust particle *d̂_i_* with index *i*, its radius *r_i_* and the number of edges *n*, the edge vertices *v_i_* of a circle-like particle can be calculated as formulated in the following equation

**(1):**
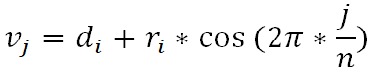


where 0 ≤ *j* ≤ *n*. These vertices are then appended to form a polygon. A similar approach is used for varying the particle shape. Setting a property value *k* ∈ [0; 2], which controls the scale of the shape variance, a new radius is calculated for each of the edge vertices by

**(2):**
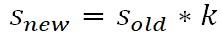


These randomly shaped particles are then smoothed by using simple interpolation between two edge points. For each particle at location di, the left and right neighbor (*d_i-1_* and *d_i+1_*) are taken into account. Finally, the edge vertices are smoothed using the equation

**(3):**
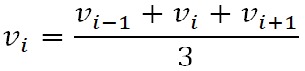


### Image composition.

Every image that enters the simulation has already been recorded with a real camera. The parameters chosen for the simulation should therefore be as close as possible to the real recording camera. The appearance of the dust particles will only yield correct results if this condition holds. For each pixel *p_x,y_*, on the output image, the following steps are performed to calculate the final pixel output color.

First, the intersection point *p_w_* of the light ray starting at the pixel at *p_x,y_*, and leaving through the center of the aperture towards the scene with the dust plane needs to be found. In case of perpendicular planes, this can be calculated rather easy using similar triangles Willson et al. ( 16 ). If the plane can have arbitrary geometry, it is best calculated using ray-plane-intersection.

Second, the projection of the collection cone at the point *p_w_* needs to be calculated. This is done by projecting the triangle edge points of the aperture onto the plane, gaining a projected polygon *c_w_* of the collection cone section with area A.

To determine the influence of the dust particles on the final output color, all surrounding particles that satisfy the condition ‖*p_w_* − *d_i_*‖ < α + 2 ∗ *r_i_* are retrieved. They are referred to as the subset *C* of dust particles in the following. These particles potentially have an influence on the final pixel color. To calculate the magnitude of that influence, for each particle *d_i_* ∈ *C*, the intersection area *A_i_* with *c_w_* is calculated. If we assume that the equation for the overall area holds:

**(4):**
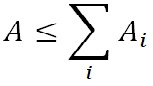


**Figure 3:**
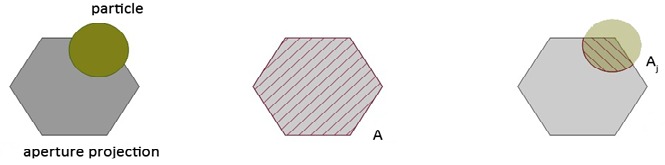
Estimation the mixing factors for the final output color. The blending factor is calculated as the fraction of each particle of the projected aperture area.

The fraction *α_i_* = *A_i_/A* is the alpha-blending factor of *d̂_i_* and determines the amount of its color contributing to the final pixel color. Therefore, if a particle has huge overlap with the current collection cone, the final output color of that pixel is strongly mixed with the particle color. Figure 3 visualizes this process.

### Optimization of the computational time

For fast retrieval of the particles close to *c_w_*, the boost implementation of an rtree is used. Further, since the blending factors are constant as long as the camera parameters remain the same, an attenuation image is calculated that can be applied to all subsequent images of a stream. The generation of the attenuation image is computationally expensive, whereas the application to an image can be done in real-time. The attenuation image contains the alpha-blending values and the color information for each pixel on the sensor and is valid as long as the camera parameters remain fixed.

### Dataset

**Figure 4:**
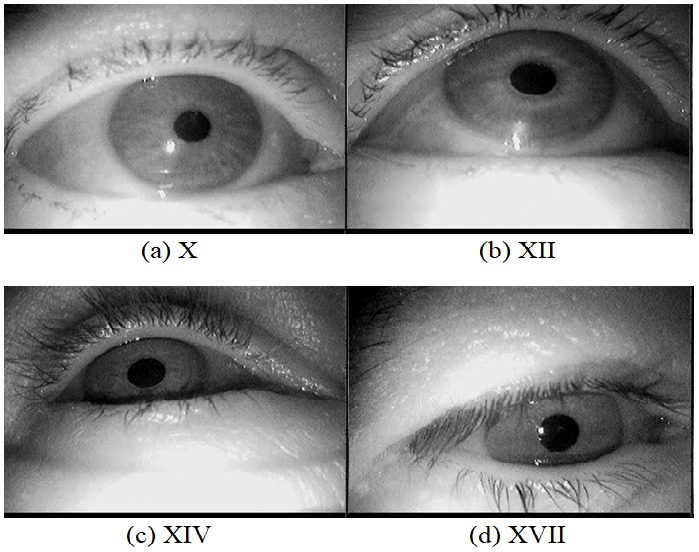
Example images selected from the respective data sets published by Fuhl et al. (2015).

We evaluated our approach on a subset of the data set by Fuhl et al. ( 2 ), namely data set X, XII, XIV, and XVII (Figure 4). Based on visual inspection, these data sets were found to be mostly free of dust particles and provided thus good baseline results for all of evaluated algorithms. A total of 2,101 images from four different subjects were extracted. These images do not contain any other challenges to the pupil detection such as make-up or contact lenses, since we wanted to investigate the iso-lated influence of dust and dirt. However, all subjects wore eyeglasses and the ambient illumination changed. Furthermore, we did not use completely synthetic images as comparable results can only be achieved within strict laboratory conditions, where dust would usually simply be removed from the recording devices.

**Figure 5:**
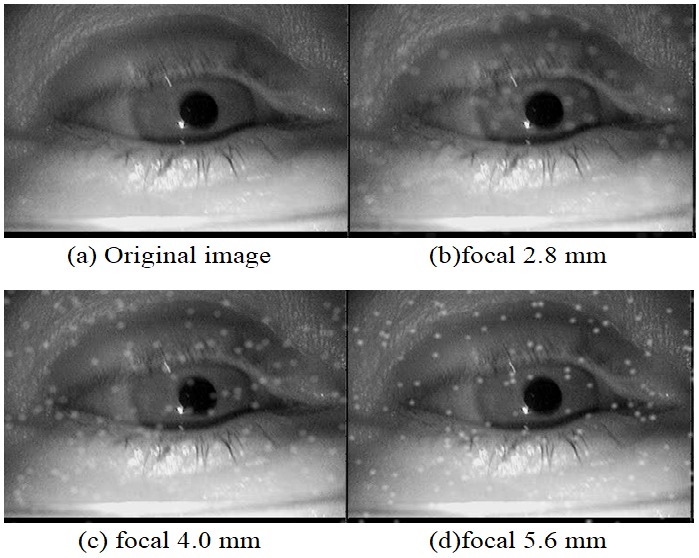
Simulation results for different focal lengths on one image. 200 particle of size group 2 were inserted.

Figure 5 shows the influence of the focal length on the final image. It should be noted here that in a realistic scenario the focal length would also have an influence on the image of the eye, not just on the particles. This effect was omitted here (visible for example at the eyelashes). For the images in Figure 5, a focal length of 5.6 puts the dust particles in focus. For real dust particles this is based on their distance to the camera and depends mainly on the design of worn glasses. Most eye cameras do not employ an autofocus mechanism but provide a possibility of adjusting the focus. However, it is rarely adjusted with dust on the eyeglasses in mind (to our experience also by the manufacturers). In the following, we will use the value of 5.6mm focus as a reference.

**Figure 6:**
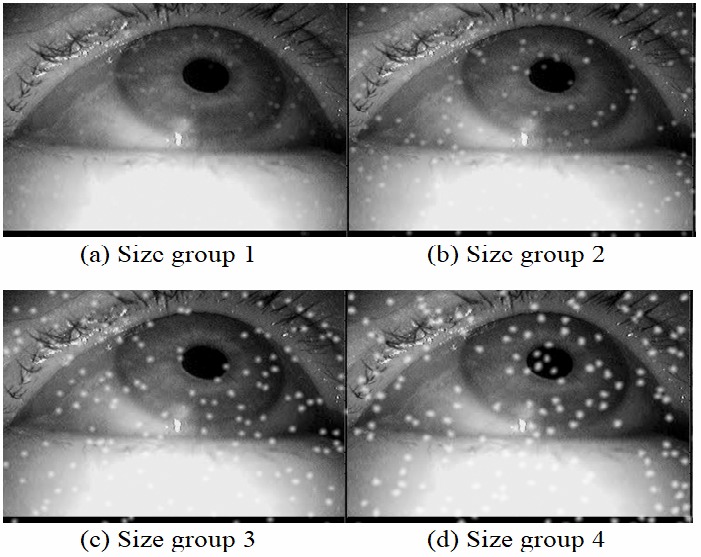
Simulation results for different particle size groups on one image. The particle amount is set to 200 and the focal length is 5.6.

Another important aspect of dirt is the size of different particles. This effect is shown in Figure 6. The amount and focal length is fixed to 200 and 5.6 respec-tively. For real world recordings dust can occur in differ-ent sizes for which we used four size groups. As can be seen in figure 6(d) they are not simple dots they are vary-ing polygons.

**Figure 7:**
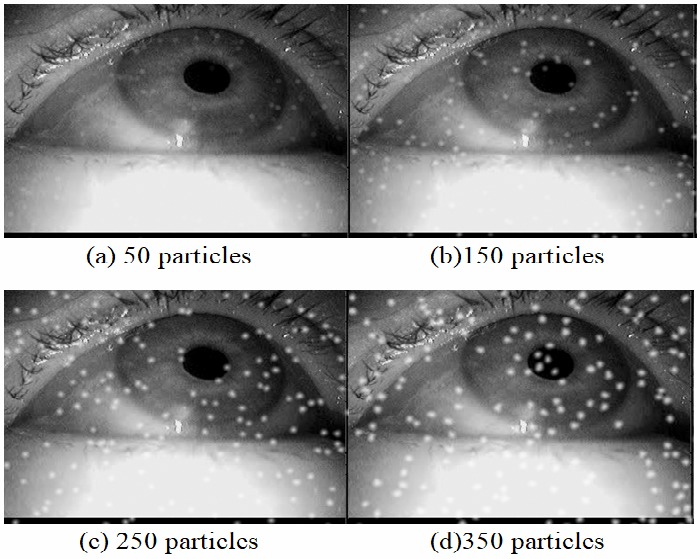
Simulation results for different amounts of particles on one image. The particle size group is set to 2 and the focal length is 5.6.

The effect of the amount of particles rendered can be seen in Figure 7. The particles are spread uniformly over the image. This is one limitation of the current simula-tion, as realistic dust distributions would include the lens lenticular buckle of the camera and the curvature of the glasses of a subject.

## Results

**Figure 8:**
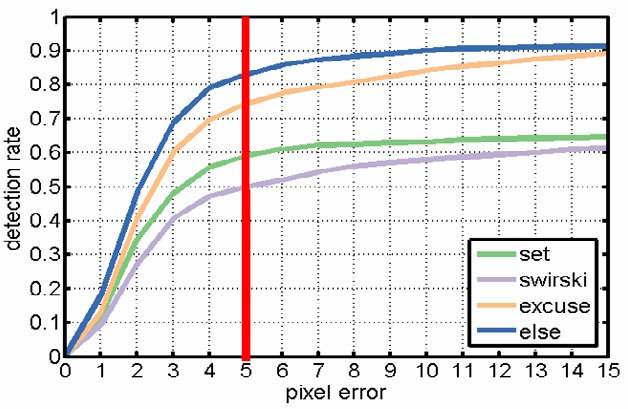
Results on all data sets without dust simulation. The detection rate is shown with regard to the Euclidean distance error in pixels. The vertical red line shows the tracking rate at a 5 pixel error, the tolerance where all algorithms have reached saturation and that we will use throughout the following evaluation.

Figure 8 shows the detection rate of the evaluated algorithms over all data sets. The detection rate is reported based on the difference in pixels between the manually labeled and the automatically detected pupil center (pixel error). The red vertical line marks the results (i.e., detection rate) for a pixel error of 5, which is considered as an acceptable pixel error for the given image resolution.

For the evaluation, we simulated the data sets with all combinations of focal length (i.e., 2.8, 4.0, and 5.6), size groups (1-4) and particle amount (50-500). Dirt particle placement is calculated based on a uniform distribution. To ensure the same dirt placement for each algorithm, we stored the simulation results and performed the evaluation on images.

Tables 1, 2, 3, and 4 show the impact of different simulation parameters on the detection rate of the algorithms SET, Świrski, ExCuSe, and Else, respectively. The provided values describe the loss of detection rate for a pixel error of five in direct comparison to the detection rate on the original data set. According to these result, the algorithm SET Javadi et al. ( 7 ) seems to be more robust to dust than the competitor algorithms. Interestingly, in Fuhl, Tonsen, et al. ( 5 ), SET was found to handle reflections inappropriately, yet in this evaluation SET showed highest robustness to simulated dirt. Only the focal length in combination with large amounts of white dust interferes with the pupil detection of this method. The reason for this robustness is related to the threshold based nature of SET.

**Table 1:** Performance of the SET algorithm. The results show the reduction in detection rate (in %) due to dirt for an error rate of 5 pixels. The baseline are detection rates achieved on clean images. F represents the focal length, SG the size group, whereas P50-P500 values specify the amount of particles. Bold highlights a reduction in the detection rate relatively to clean data of more than 10%.

F	SG	P50	P100	P200	P300	P400	P500
2.8	1	0	0	-2	-2	-2	-3
	2	0	-2	-7	-9	**-12**	**-13**
	3	-3	-1	**-12**	**-17**	**-29**	**-24**
	4	-3	**-13**	**-24**	**-22**	**-37**	**-52**
4.0	1	0	1	-1	-2	-1	-2
	2	0	0	-2	-3	-4	**-11**
	3	-1	-6	-7	-9	-9	**-20**
	4	-1	-3	-6	**-12**	**-20**	**-36**
5.6	1	0	-1	-1	-2	-3	-2
	2	-1	0	-3	-1	-5	-5
	3	0	-2	-9	-8	-8	**-14**
	4	2	-2	-8	**-18**	**-18**	**-21**

**Table 2:** Performance of the Świrski algorithm. The results show the reduction in detection rate (in %) due to dirt for an error rate of 5 pixels. The baseline are detection rates achieved on clean images. F represents the focal length, SG the size group, whereas P50-P500 values specify the amount of particles. Bold highlights a reduction in the detection rate relatively to clean data of more than 10%.

F	SG	P50	P100	P200	P300	P400	P500
2.8	1	-3	-2	-2	-4	-2	-4
	2	-3	-5	**-23**	**-25**	**-16**	**-33**
	3	**-12**	**-18**	**-36**	**-48**	**-58**	**-43**
	4	**-23**	**-42**	**-46**	**-44**	**-55**	**-65**
4.0	1	-1	-4	-5	**-14**	-2	-3
	2	-3	-3	**-14**	**-15**	**-17**	**-31**
	3	-8	**-29**	**-36**	**-39**	**-60**	**-52**
	4	-8	**-37**	**-36**	**-42**	**-46**	**-69**
5.6	1	-2	-1	-5	-1	-5	-2
	2	0	0	**-14**	-4	**-14**	**-13**
	3	-1	**-14**	**-25**	**-42**	**-29**	**-60**
	4	-9	**-25**	**-56**	**-44**	**-58**	**-66**

**Table 3:** Performance of the ExCuSe algorithm. The results show the reduction in detection rate (in %) due to dirt for an error rate of 5 pixels. The baseline are detection rates achieved on clean images. F represents the focal length, SG the size group, whereas P50-P500 values specify the amount of particles. Bold highlights a reduction in the detection rate relatively to clean data of more than 10%.

F	SG	P50	P100	P200	P300	P400	P500
2.8	1	2	1	2	1	1	0
	2	0	-1	-2	-7	**-11**	**-14**
	3	-2	-7	**-14**	**-27**	**-35**	**-37**
	4	-4	**-17**	**-31**	**-45**	**-42**	**-59**
4.0	1	2	1	1	0	-1	-2
	2	0	-5	**-12**	**-17**	**-25**	**-28**
	3	-5	**-11**	**-26**	**-26**	**-57**	**-54**
	4	-6	**-18**	**-29**	**-47**	**-62**	**-75**
5.6	1	0	0	-2	-5	-5	-8
	2	-3	-8	**-20**	**-25**	**-40**	**-42**
	3	-5	**-16**	**-31**	**-48**	**-53**	**-76**
	4	**-13**	**-25**	**-44**	**-58**	**-71**	**-83**

**Table 4:** Performance of the ElSe algorithm. The results show the reduction in detection rate (in %) due to dirt for an error rate of 5 pixels. The baseline are detection rates achieved on clean images. F represents the focal length, SG the size group, whereas P50-P500 values specify the amount of particles. Bold highlights a reduction in the detection rate relatively to clean data of more than 10%.

F	SG	P50	P100	P200	P300	P400	P500
2.8	1	0	0	0	-1	-2	-1
	2	-2	-4	-5	-8	**-12**	**-14**
	3	-3	-7	**-13**	**-21**	**-31**	**-33**
	4	-5	**-15**	**-31**	**-40**	**-43**	**-59**
4.0	1	0	-1	-2	-3	-3	-5
	2	-3	-9	**-14**	**-29**	**-29**	**-31**
	3	-8	**-13**	**-30**	**-54**	**-54**	**-51**
	4	**-10**	**-22**	**-33**	**-61**	**-61**	**-79**
5.6	1	-2	-2	-5	-8	-8	**-11**
	2	-4	-8	-2	**-28**	**-40**	**-38**
	3	-4	**-14**	**-29**	**-43**	**-50**	**-70**
	4	**-13**	**-21**	**-41**	**-54**	**-69**	**-77**

The algorithms Świrski et al. ( 12 ), ExCuSe Fuhl et al. ( 2 ) and ElSe Fuhl, Santini, Kübler, and Kasneci ( 4 ) are, in contrast, all based on edge detection. Since dust particles in the image interfere with the performance of the Canny edge detector, the pupil boundary cannot be extracted robustly. In addition, the induced edges by the particles themselves connected to the pupil edge make the ellipse fit much harder. Therefore, further improvements to the algorithms should inspect automatic threshold adjustments. In addition, preliminary image refinement steps are necessary since thresholding is not appropriate for light gradients over the pupil.

**Figure 9:**
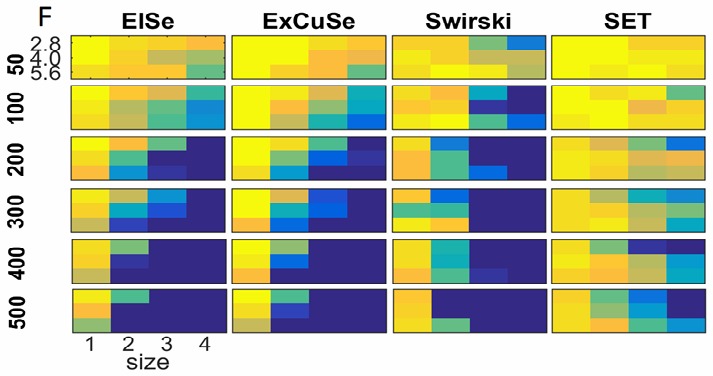
Heatmap visualization of the results shown in Tables 1-4. The chosen colors reach from yellow over green to blue, where yellow stands for no influence on the algorithmic performance, whereas dark blue represents highest negative influence on the performance of the pupil detection.

For a better overview, a heatmap visualization of the results given in Tables 1-4 is shown in Figure 9. In this visualization, yellow represents lowest influence on the algorithm performance, whereas dark blue represent highest negative influence on the algorithmic performance.

## Discussion

We proposed a dirt simulation and evaluation for eye images as obtained by commercially available eyetrackers. Such a simulation can help to evaluate algorithms regarding their applicability in the wild and to explore their limitations. Besides simulating different colors and particle sizes for dirt, our approach offers the possibility to vary the focal length, which could also happen in real scenarios since the automatic focus estimation is influenced by the dirt layer. We found most algorithms to be relatively robust towards few large off-focus dust particles. These are particles close to the camera lens (or for example a glass cover when the camera is mounted within a car dashboard). We can therefore conclude that the amount of dust that can be tolerated on the tracking device itself is quite large, given the right choice of pupil detection algorithm.

Overall, it has to be mentioned that lower detection rates of individual algorithms also impact the tracking loss significantly. While the edge-based pupil localization methods still outperformed threshold-based methods for most of the simulations, even small in-focus dust particles can result in a huge impact on their performance. However, this impact is likely occurring in images where the pupil is hard to detect, so that other methods already failed at the baseline level and show therefore only a smaller percentual loss. This finding highlights (i) that the current generation of pupil detection algorithms are vulnerable to dust particles and (ii) the importance of a sharp and intelligent autofocus for head-mounted trackers in order to select the actual eye depth layer instead of the eyeglasses as accurately as possible.

This work provides a task-plan for the further improvement of pupil edge-based pupil localization methods that should focus on automatic threshold range adjustments, image refinement, and reconstruction (dirt removal and filtering). Dirt particles are static on the subject’s glasses and can therefore be identified in a video sequence. Removing this noise factor could improve the algorithmic performance and robustness.

In future work, we will evaluate our simulation results against real dirt conditions on the camera and on subject’s glasses. In addition, we will investigate the robustness of pupil detection algorithms based on deep neural networks, such as PupilNet Fuhl, Santini, Kasneci, and Kasneci ( 3 ), to dust. Further improvements to the simulation itself will include a combination with the synthesis module for glasses from Kübler et al. ( 9 ) and inclusion of different dirt area distributions.

## Ethics and Conflict of Interest

The author(s) declare(s) that the contents of the article are in agreement with the ethics described in http://biblio.unibe.ch/portale/elibrary/BOP/jemr/ethics.html and that there is no conflict of interest regarding the publication of this paper.

## Acknowledgements

We acknowledge support by Deutsche Forschungsgemeinschaft and Open Access Publishing Fund of University of Tübingen.
